# Ensilage and Disease

**Published:** 1881-04

**Authors:** 


ENSILAGE AND DISEASE.—The subject of ensilage is now agitating the whole agricultural world. Ensilage is, essentially, corn-fodder, cut up, packed in air-tight pits, and allowed to remain there while it undergoes to some extent the acetic acid (?) fermentation. By this process, it is said that the cost of feeding cattle is reduced from one-half to one-fourth.

The changes produced by ensilage result in keeping the water in the corn and in diminishing the amount of non-nitrogenous matter. In some cases a great acidity of the fodder is produced. Cattle generally eat the new food with great avidity, and it seems to agree with them.

We advise veterinarians, however, to be on the watch. It is said that with every new invention there comes a new disease. This generalization, if ever true, should apply in the case of ensilage, which involves a great change in the diet. We are told that ensilage fodder makes rich milk, and preserves the animal in good condition. But we have yet to see whether it does not eventually produce some digestive or other disturbances; whether it does not affect the flesh, or the productiveness of the animal, or the health of its offspring. Bad effects, indeed, are already being announced by some. It will certainly be an interesting fact if so extensive a modification of diet does not in some way show itself on the animal. And even if cattle are unaffected, it must still be learned whether the horse thrives equally well. A modification of ensilage has for a long time been employed in Germany, but not so extensively as to furnish accurate data for judging upon the merits of the system now being popularized in this country.

